# Facile approach to fabricate waterborne polyaniline nanocomposites with environmental benignity and high physical properties

**DOI:** 10.1038/srep43694

**Published:** 2017-03-06

**Authors:** Haihua Wang, Huan Wen, Bin Hu, Guiqiang Fei, Yiding Shen, Liyu Sun, Dong Yang

**Affiliations:** 1 Key laboratory of Auxiliary Chemistry & Technology for Chemical Industry, Ministry of Education, Shaanxi University of Science and Technology, Xi’an, Shaanxi, 710021, China; 2 Wuhan National Laboratory for Optoelectronics (WNLO), School of Optical and Electric Information, Huazhong University of Science and Technology (HUST), Wuhan 430074, PR China

## Abstract

Waterborne polyaniline (PANI) dispersion has got extensive attention due to its environmental friendliness and good processability, whereas the storage stability and mechanical property have been the challenge for the waterborne PANI composites. Here we prepare for waterborne PANI dispersion through the chemical graft polymerisation of PANI into epichlorohydrin modified poly (vinyl alcohol) (EPVA). In comparison with waterborne PANI dispersion prepared through physical blend and *in situ* polymerisation, the storage stability of PANI-g-EPVA dispersion is greatly improved and the dispersion keeps stable for one year. In addition, the as-prepared PANI-g-EPVA film displays more uniform and smooth morphology, as well as enhanced phase compatibility. PANI is homogeneously distributed in the EPVA matrix on the nanoscale. PANI-g-EPVA displays different morphology at different aniline content. The electrical conductivity corresponds to 7.3 S/cm when only 30% PANI is incorporated into the composites, and then increases up to 20.83 S/cm with further increase in the aniline content. Simultaneously, the tensile strength increases from 35 MPa to 64 MPa. The as-prepared PANI-g-EPVA dispersion can be directly used as the conductive ink or coatings for cellulose fibre paper to prepare flexible conductive paper with high conductivity and mechanical property, which is also suitable for large scalable production.

Polyaniline (PANI) has got special attention amongst conductive polymers owing to the adjustable conductivity, easy preparation, good environmental stability and unique doping/dedoping mechanism. It has been extensively applied in various fields^1^, such as portable and wearable/foldable electronics^2^, capacitor^3^,^4^, electromagnetic anticorrosion coatings^5^, shielding devices^6^, electrochromic displays^7^, static electricity dissipation^8^, and so on. However, its applications were restricted due to its poor solubility, infusibility, poor processability and low mechanical properties^a^^9^. Therefore, various strategies have been put forward. Improving water dispersibility of PANI has been demonstrated as a approach to improve the processability of PANI. In particular, it is advantageous to the environment protection^10^,^11^. The incorporation of appropriate substitutes, such as sulfonic acid, boronic acid and carboxylic groups, at the phenyl rings or nitrogen sites of PANI has turned out to be a simple and effective methodology to improve the solubility of PANI^12^,^13^,^14^. In addition, waterborne PANI dispersion with good stability could be prepared through *in situ* synthesis of PANI using cellulose nanocrystals or graphene oxide^15^,^16^. The copolymerisation of aniline derivatives and aniline is also an alternative approach to prepare self-doped and water-soluble PANI derivative^10^,^17^. Morsi *et al*.^10^ synthesised self-doped PANI by using aniline and 4-amino benzenesulfonic acid as monomers. Moreover, surfactants are also utilized for the stabilisation of waterbornePANI and polypyrroledispersion, such as poly (*N*-vinylpyrrolidone), methylcellulose, and polyacrylic acid^2^,^18^,^19^,^20^. The character of surfactant is crucial to control the dispersion and morphology of nanocomposites^21^,^22^.

Although the processability of PANI can be improved by increasing the dispersibility of PANI, the materials made from waterborne PANI dispersions still have defects in mechanical properties and adhesion^23^. Several approaches have been developed to overcome such defects. For instance, polymer emulsion has been adopted as the matrix to blend with waterborne PANI dispersion to combine the electroactivity of PANI with physical properties of polymer emulsion. Santos *et al*. obtained waterborne PANI composites by mixing PANI with 10% natural latex andaqueousphytic acid was utilised as dopant, the adhesion and corrosion resistance of composite coating are significantly improved^24^. On the other hand, electrochemical polymerisation of aniline in aqueous polymer solution is also a good approach^25^. In order to further increase the compatibility between PANI and polymer matrix, core-shell emulsion polymerisation^26^,^27^ and *in situ* polymerisation^2^,^28^,^29^ are put forward.

As a polymer matrix, polyvinyl alcohol (PVA) has attracted more attention because of its advantages including non-toxicity, water-solubility, good chemical stability, toughness and adhesion property, film-forming property and possible coupling of charge transport with the motion of its hydroxyl groups^30^,^31^. Moreover, PVA is able to simultaneously functionalise as polymeric stabilizer, co-dopant and matrix^31^,^32^,^33^,^34^. Arenas *et al*.^35^ prepared aqueous suspensions of PVA–PANI nanocomposites by conventional polymerisation of aniline in the presence of PVA solution mixed with either surfactant(sodium dodecyl sulfate) or organic acids used as binary dopants, to improve the solubility and processability of the nanocomposites. Mirmohseni *et al*.^33^ prepared the polyaniline composite films by chemical polymerization of aniline in the media containing polyvinyl alcohol. The electrical conductivity of the films greatly increased to 2.5 S/cm with the increase of PANI amount. However, the composite property was impaired due to the presence of hydrophilic groups. Partially phosphorylated poly(vinyl alcohol) (P-PVA), as a phosphoric ester by phosphorylation of PVA with phosphoric acid, exhibits more effective stabilisation, higher water resistance and mechanical property. Chen and Liu^36^ prepared PANI/P-PVA dispersions by the chemical oxidative polymerisation of aniline in aqueous acidic media containing the P-PVA, which display significant dispersibility in aqueous media. Waterborne epoxy coating containing PANI/P-PVA possesses excellent corrosion protection property. In addition, PVA conjugated with 2-isobutyramidopropanoate (PVA-AI) was also reported as dispersing agent to improve the dispersibility and anticorrosion property of PANI/PVA^37^. Varakirkkulchai *et al*.^38^ obtained P-PVA-PANI/polyacrylate nanoparticles by encapsulating P-PVA-PANI with polyacrylate via emulsifier-free emulsion polymerisation. However, most of researches focus on the physical blend of PVA and PANI and *in situ* oxidation polymerisation of aniline in PVA solution, and very few studies investigated chemical graft modification to improve the dispersibility and distribution.

Herein, we report for the first time a facile approach to fabricate an environmental waterborne polyaniline nanocomposite through the chemical graft of PANI into epoxy modified PVA (EPVA) based on the reaction between epoxy groups in PVA and amine groups in PANI, and the epoxy groups are introduced into PVA chain through nucleophilic substitution with epichlorohydrin (ECIP). The molecular design not only inhibits the precipitation of PANI from waterborne polymer matrix and correspondingly improves the stability of waterborne PANI-graft-EPVA dispersion thanks to the formed chemical bond between PANI and EPVA, but also ensures the homogeneous distribution of PANI in PANI-g-EPVA nanocomposites and thereby forms 3D interconnected conducting network with uniformly distributed PANI. The conductivity of PANI-g-EPVA film increases up to 20.83 S/cm with increasing the aniline content to 45%. In general, the conductivity of conventional emeraldine. HCl PANI^3^ is about 2–10 S/cm, whereas the conductivity of PANI-g-EPVA nanocomposite reaches7.3 S/cm with the addition of 30% aniline. The tensile strength increases from 35 MPa to 64 MPa. The as-prepared PANI-g-EPVA dispersion can be directly used as the conductive ink or coatings for cellulose fibre paper to prepare flexible conductive paper with high conductivity and mechanical property.

## Results and Discussion

### Comparison between *in situ* PVA/PANI and PANI-g-EPVA

**Figure 1:**
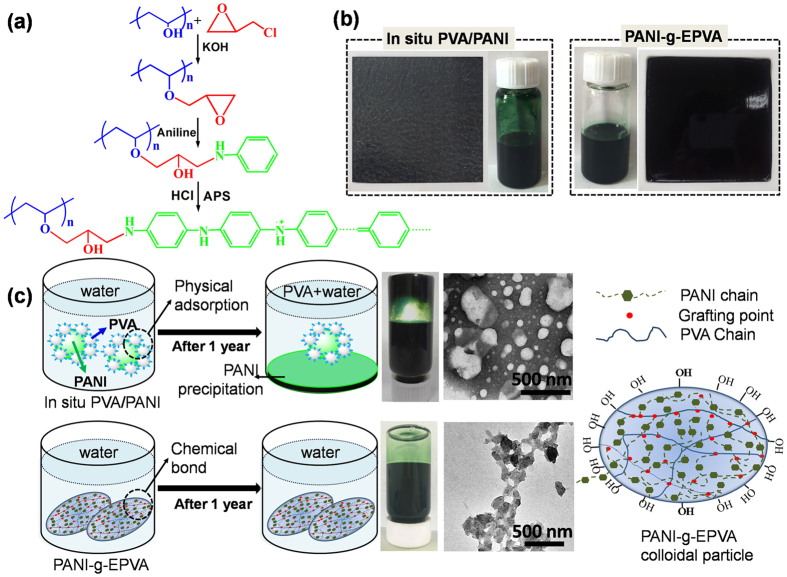
(**a**) Preparation scheme of PANI-g-EPVA. (**b**) The optical photos of *in situ* PVA/PANI and PANI-g-EPVA dispersions and the corresponding films. (**c**) The molecular model and stabilizing mechanism of *in situ* PVA/PANI and PANI-g-EPVA dispersions, as well as the optical photos and TEM morphology.

In this study, we prepare PANI-g-EPVA nanocomposites through chemical graft polymerisation, the preparation scheme is shown in Fig. 1a. In order to make comparison between conventional preparation method and chemical graft polymerization, *in situ* PVA/PANI nanocomposite is also prepared through the oxidation polymerisation of aniline in PVA solution. The optical photos of *in situ* PVA/PANI and PANI-g-EPVA dispersions and the corresponding films are presented in Fig. 1b. It is found that PANI-g-EPVA film displays more uniform and smooth morphology compared with *in situ* PVA/PANI film. And macroscopic PANI precipitation was initially generated inside *in situ* PVA/PANI dispersion after storage for 15 days, and the amount of PANI precipitation increases with prolonging the storage time. In contrast, the PANI-g-EPVA dispersion keeps stable for more than one year, as presented in Fig. 1c. Particle agglomerations are detected in the TEM morphology of *in situ* PVA/PANI dispersion, and the particle size distributes unevenly. While the particle size of PANI-g-EPVA dispersion is more uniform.

With respect to the chemical graft modification, chemical bonds are built between the PANI and PVA (as shown in the molecular model of PANI-g-EPVA colloidal particles in Fig. 1c), which is favourable to prohibit the PANI aggregation and phase separation. However, as to *in situ* PVA/PANI, PVA functionalises as stabiliser^30^,^39^, PANI oligomers are firstly produced in the early stage of aniline oxidation, and then adsorb at PVA chains. Once the first PANI chain is produced at the stabiliser chain, the aniline oxidation will proceed in close vicinity^40^. In order to keep the dispersion stable, the hydrophilic PVA stabiliser tends to distribute at the particle surface to form the shell of colloidal particles, and prevents the PANI agglomeration, as shown in Fig. 1c. However, PANI agglomerations take place when the PANI content exceeds certain amount, i.e. the amount of PVA is not high enough to encapsulate the PANI. In addition, the interaction between PANI and PVA is mainly dominated by physical interaction in *in-situ* PVA/PANI. Phase separation tends to take place as prolonging the storage time, resulting in the agglomeration of PANI, as illustrated in Fig. 1c. Therefore, it can be concluded that building chemical bonds between PANI and other polymer matrix is an effective approach to improve the dispersibility and distribution of PANI in waterborne polymer dispersions, as well as the storage stability of waterborne PANI-g-EPVA dispersion.

**Figure 2:**
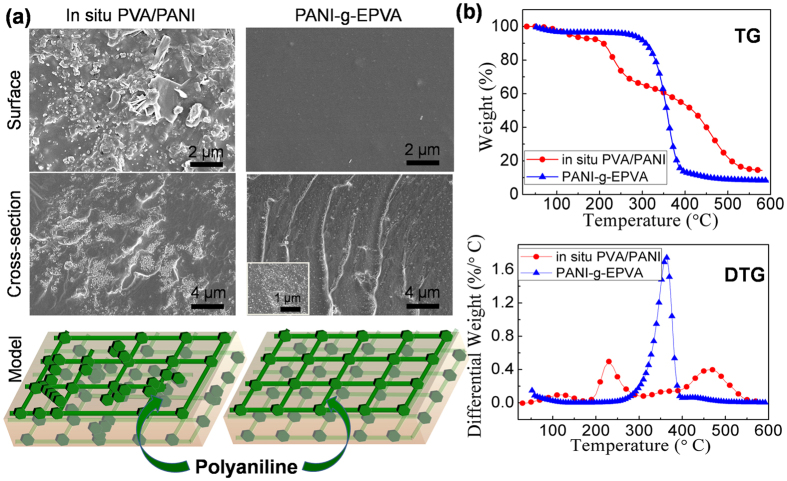
(**a**) The surface and cross-section SEM morphology and representative model of *in situ* PVA/PANI and PANI-g-EPVA. (**b**) TG and DTG curves of *in situ* PVA/PANI and PANI-g-EPVA.

Figure 2a shows the surface and cross-section morphology of *in situ* PVA/PANI and PANI-g-EPVA nanocomposite films with 30% aniline content. It is also obvious that PANI homogenously distributes inside the PANI-g-EPVA nanocomposite, while PANI coagulation is observed for *in situ* PVA/PANI. It further certifies that chemical graft modification is able to improve the dispersibility and distribution of PANI in polymer matrix, in comparison with conventional *in situ* polymerisation.

TG-DTG curves can provide further information on the phase behaviour between PANI and PVA for *in situ* PVA/PANI and PANI-g-EPVA, as illustrated in Fig. 2b. It is found that *in situ* PVA/PANI degrades in three steps. The first stage at 85–176 °C is mainly attributed to the removal of water and undopedHCl^30^. The second stage at 176–307 °C is the superposition of the elimination of side-groups, the H-boned water and the covalent bonds of PVA. The third stage at 360–570 °C is mainly the breakage of the main chains of both polymers (EPVA and PANI)^30^. However, PANI-g-EPVA shows different degradation behaviours, the two main degradation peaks merge together and only one degradation peak at 362 °C is detected. This suggests good compatibility between PANI and EPVA, and the graft of PANI into PVA chains retards the degradation of PVA chains.

### Structure and morphology analysis

The FTIR spectra (see Supplementary Fig. S1) and ^1^H-NMR spectra (see Supplementary Fig. S2) of PVA, EPVA, EPVA-aniline and PANI-g-EPVA were provided to demonstrate each reaction step in the preparation process of PANI-g-EPVA. In the FTIR spectrum of PVA, the peaks at 3315 cm^−1^, 2927 cm^−1^, 1716 cm^−1^ and 1076 cm^−1^ are due to the stretching vibration of –OH, –CH_2_, C = O and C-O, respectively^30^,^35^. A novel peak at 913 cm^−1^ is observed for EPVA after the nucleophilic substitution with epichlorohydrin, which is corresponding to the characteristic adsorption peak of epoxy group. It suggested that the epoxy groups are successfully incorporated into the PVA chains. The absence of peak at 1716 cm^−1^ indicates the hydrolysis of ester groups during the process of nucleophilic substitution reaction. The peak at 1664 cm^−1^ in EPVA spectrum can be ascribed to the deformation vibration of -OH. With the introduction of aniline, the peak at 913 cm^−1^ corresponding to the adsorption of epoxy groups almost disappeared, certifying the reaction between aniline and EPVA. With the incorporation of PANI, the intensity of -OH adsorption peak decreases, and the peak value shifts to lower wavenumber at 3240 cm^−1^ due to overlap of the residual -OH groups in EPVA and -NH groups in PANI^1^,^30^. The novel peaks at 1656 and 1582 cm^−1^ are corresponding to the C = C stretching vibration peak of benzene ring and quinone ring in PANI molecule and the C = N stretching vibration peak of quinone ring^41^. The peaks about 1062 cm^−1^ is assigned to stretching absorption peaks of in-plane-bending vibration of C–H (mode of N = Q = N, Q = N^+^H-B, and B-N^+^H-B; Q = quinoid ring, B = benzenoidring)^41^,^42^. The peaks at 1292 cm^−1^ and 1418 cm^−1^ are corresponding to the C-N bond stretching vibration in PANI^42^.

The ^1^H-NMR spectra of the PVA, EPVA, EPVA-aniline and PANI-g-EPVA are also provided, as illustrated in Supplementary Fig. S2. In the spectrum of PVA, the triplet signals from 4.19 ppm to 4.65 ppm are attributed to the proton of –OH, which can be attributed to the three spatial stereoregularity of PVA^40^. With the introduction of epoxy groups, a novel signal appeared at 3.37 ppm, which can be ascribed to the proton of -CH in oxirane^43^. The signal around 1.77 ppm (-COOCH_3_) disappeared due to the hydrolysis of ester groups in this step, which is consistent with the FTIR results. In the NMR spectrum of EPVA-aniline, the signals at 7.3–6.4 ppm allow estimation of the presence of aromatic proton signals of aniline^44^. Then the peak almost disappears as the oxidation polymerization of aniline proceeds. The signals at 3.81 ppm and 6.90–7.31 ppm are the proton of -NH and quinone ring, corresponding to the characteristic adsorption peaks of PANI^45^. The above results certify that PANI is successfully grafted into PVA matrix and the entire preparation process of PANI-g-EPVA.

The graft efficiency (GE) of PANI-g-EPVA nanocomposites was also determined by elemental analysis. GE value is 52.5%, 48.66%, 41.90% when the aniline content was 20%, 30% and 40%, respectively (see Supplementary Table S1). It indicates that aniline monomers tend to self-polymerization and form free PANI in the PANI-g-EPVA nanocomposite with increasing the aniline content. The PANI-g-EPVA nanocomposite is probably composed of PANI-g-EPVA, free PANI and free EPVA. The introduction of graft copolymer is beneficial to improve the stability and compatibility between PVA and PANI. The FTIR spectra (see Supplementary Fig. S3) and ^1^H-NMR spectra (see Supplementary Fig. S4) of PANI-g-EPVA prepared with different aniline content are also provided. The intensity of peaks at 1582 cm^−1^ and 1292 cm^−1^ corresponding to the quinone ring and C-N bond stretching vibration in PANI increases when increasing the aniline content from 20% to 30%, and keeps almost invariable with increasing the aniline content from 30% to 40%. The intensity of PANI characteristic signals also displays similar trend as FTIR spectra. It means that the grafted amount of PANI in EPVA increases and then keeps almost constant with increasing aniline content, resulting in the decrease of GE.

**Figure 3:**
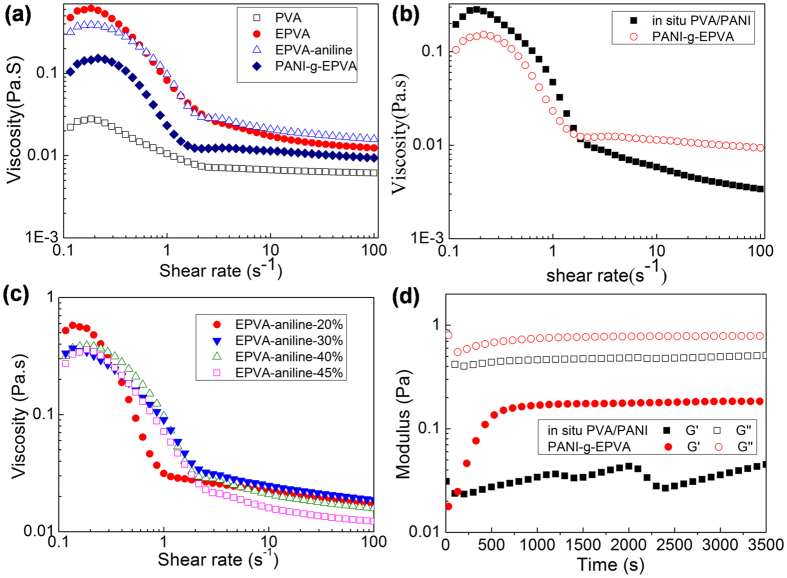
(**a**) Steady rheology of PVA, EPVA, EPVA-aniline and PANI-g-EPVA corresponding to each preparation step. (**b**) Steady rheology of *in situ* PVA/PANI and PANI-g-EPVA. (**c**) Steady rheology of EPVA-aniline at different aniline content. (**d**) Variation of modulus with time for *in situ* PVA/PANI and PANI-g-EPVA at 80 °C.

In order to further investigate the preparation process and the structure of PANI-g-EPVA dispersion, rheological behaviours of PVA, EPVA, EPVA-aniline and PANI-g-EPVA at each preparation step are studied, as well as the *in situ* PVA/PANI and PANI-g-EPVA dispersion prepared at different aniline content, as shown in Fig. 3. Rheology has been considered as efficient tool for exploring structural properties and molecular interactions of different materials viz polymer, surfactant, supramolecule etc., in solution, dispersion, stabilized colloid or gel^46^. Figure 3a shows that the viscosity of EPVA and EPVA-aniline increases with the incorporation of epoxy and aniline groups into PVA chains, which can be ascribed to increased molecular interactions. As increasing the aniline content, the viscosity of EPVA-aniline increases to a certain extent, followed by slight decrease, as shown in Fig. 3c. This suggests that the amount of grafted aniline reaches maximum when the aniline content is 30%, and self-polymerization of aniline monomers tends to take place when aniline content increases to 40%, resulting in the decrease in the amount of grafted aniline in PVA chains. Moreover, the viscosity of PANI-g-EPVA decreases after the oxidation polymerization of aniline owing to the variation in the conformation of polymer chains. As the reaction proceeds, entangled or stretched polymer chains gradually transfer to spherical micelles due to the increase of polymer hydrophobility. The rheology of PANI-g-EPVA is mainly dominated by the interactions among colloidal particles rather than the interactions among polymer chains, and the interaction points among colloidal particles are inevitably decreased in comparison with that among polymer chains, resulting in the decrease in the viscosity.

Figure 3b displays the steady rheological behaviours of *in situ* PVA/PANI and PANI-g-EPVA dispersions prepared with 30% aniline content. Although dispersions contain colloidal particles instead of polymers, if we assume the dispersed water droplets slip past each other like the reptation motion of polymer chains the results of polymer analysis can be transferred to dispersions. It is observed that both dispersions present pseudoplastic liquid behaviour^47^. The viscosity of *in situ* PVA/PANI is higher at lower shear rate and then keeps decreasing with shear rate. While the viscosity of PANI-g-EPVA decreases firstly and then keeps invariable when the shear rate is greater than 1 s^−1^. It suggests that the internal network inside *in situ* PVA/PANI dispersion is unstable compared with PANI-g-EPVA dispersion. This result is also further demonstrated by the oscillatory rheology test, as presented in Fig. 3d. Generally, storage modulus quantifies the mechanical properties of the respective liquids, therefore, it is a measure of the colloidal interactions induced by internal network inside dispersion. With respect to stable dispersion, the interactions among colloidal particles should be able to resist the gravitational force. As presented in Fig. 3d, the storage modulus of *in situ* PVA/PANI is lower than that of PANI-g-EPVA, and keeps changing due to its unstable inner network structure.

**Figure 4:**
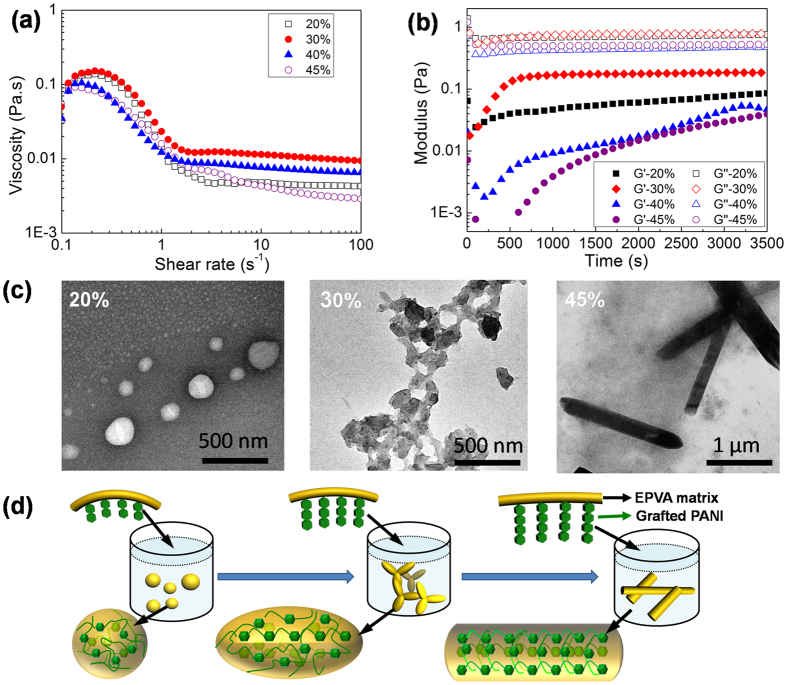
(**a**) Effects of aniline content on the steady rheology of PANI-g-EPVA dispersions. (**b**) Variation of modulus with time for PANI-g-EPVA dispersions prepared with different aniline content. (**c**) TEM images and (**d**) Schematic models of the nanostructured PANI-g-EPVA dispersion with different aniline contents.

Effects of aniline content on the rheological behaviours of PANI-g-EPVA dispersions were also investigated, as shown in Fig. 4a. With increasing the aniline content from 20% to 30%, the viscosity increases firstly and then decreases when the aniline content reaches 45%. It suggests that the interaction between colloidal particles increases with the incorporation of 30% PANI. However, the interaction is weakened with further increase in the PANI content. At lower aniline content, hydrophobic PANI tends to distribute inside colloidal particles to keep dispersion stable, the surface of colloidal particle is mainly composed of PVA components. With increasing the aniline content, some PANI chains start to distribute on the particle surface and form connected network in the dispersion, leading to the increase of viscosity. However, phase separation takes place when the aniline content increases to 40% owing to the increasing amount of free PANI inside the reaction system, which may be responsible for the decrease of viscosity. Isothermal time dependence of storage modulus (G′) and loss modulus (G″) at 80 °C with constant frequency for PANI-g-EPVA dispersions prepared with different aniline content is shown in Fig. 4b. The storage modulus of PANI-g-EPVA with 30% aniline content is the highest, and the ratio of G” to G′ is the lowest, suggesting that PANI-g-EPVA with 30% aniline content behaves more elastically than the other two dispersions. In contrast, the storage modulus of PANI-g-EPVA with 45% aniline content is the lowest, which may be not enough to resist the gravitational stress and therefore cause particle agglomeration. In addition, the storage modulus increases with prolonging the test time. which can be due to the rearrangement of the rod-like colloidal particles.

The TEM images of the PANI-g-EPVA nanocomposites prepared with different aniline contents in the polymerisation are also provided, as shown in Fig. 3. In agreement with rheological behaviour, the colloidal particles interconnect with each other when the aniline content is 30%, resulting in the increase in viscosity. The spherical particles are produced when the aniline content is 20%, the coral-like particles are obtained when the aniline content is 30%, andthe rod-like morphology is formed when the aniline content is 45% (Fig. 4c). TEM results indicate that the morphology of PANI-g-EPVA is directly related to the aniline content. The reactive points in EPVA is settled in this research, the molecular weight of PANI grafted into the EPVA increases as increasing the aniline content (Fig. 4d). At lower aniline content, the EPVA can effectively distributed on the surface of the particles, leading to the formation of spherical shapes. With increasing the aniline content, the molecular weight of grafted PANI increases, and the rigidity of PANI chain is higher than that of EPVA, the mobility of EPVA backbone is thereby restricted to a certain extent, thus coral-like particles are formed by aggregation of particles from their reactive sites. When the aniline content increases to 45%, PANI-g-EPVA chains mainly display rigid characteristic, it is very difficult for the molecular chainsto move and bend, and therefore rod-like particles are obtained through self-assembly of PANI-g-EPVA chains. The schematic models for the formation of PANI-g-EPVA nanocomposites with different morphology are shown in Fig. 4c.

Dynamic light scattering data also show that the average particle diametre (D) increases from 161.7 nm to 1533 nm with increasing the aniline content from 20% to 45%, as illustrated in Supplementary Fig. S5. The particle size distribution changes from unimodal distribution to bimodal distribution when the aniline content is greater than 30%. It can be attributed to different colloidal particles formed by PANI-g-EPVA and PANI self-aggregations, respectively. This phenomena is consistent with TEM results.

### Conductivity, thermal and mechanical property

**Figure 5:**
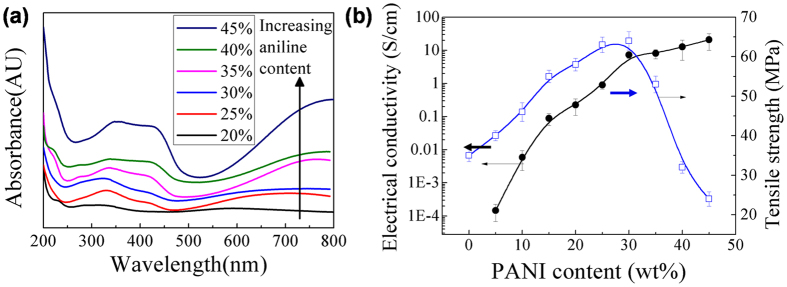
(**a**) UV-Vis spectra of the PANI-g-EPVA dispersions. (**b**) Effects of aniline content on the conductivity and tensile strength of PANI-g-EPVA nanocomposite films.

UV-Vis absorption spectra of PANI-g-EPVA dispersion with different aniline content are shown in Fig. 5a. Three characteristic adsorption bands for doped PANI at wavelength of 300–370 nm, 410–460 nm and 650–800 nm are observed. The absorption band at 300–370 nm is due to the π-π* transitions of benzene ring^48^,^49^. The absorption bands at 410–460 nm and 650–800 nm are related to the doping level and polaron-bipolaron transition^1^,^49^,^50^. The intensity of absorption bands increases with increasing the aniline content, and the polaron-bipolaron transition band exhibits a red shift, suggesting the increase in conductivity^1^.

**Figure 6:**
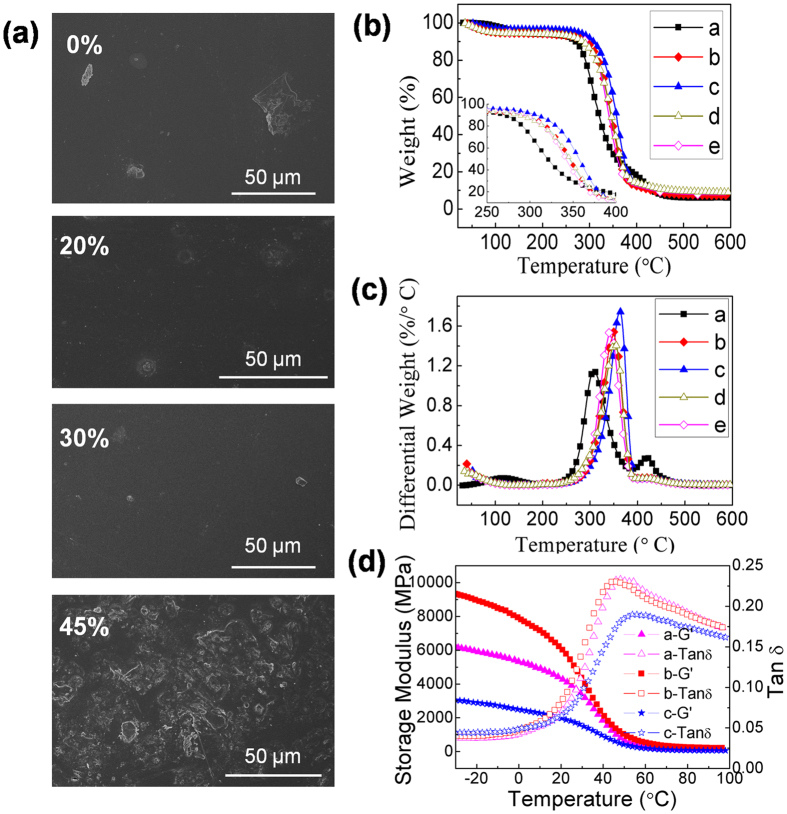
(**a**) SEM photographs of the PANI-g-EPVA nanocomposite films at different aniline contents. (**b**) TG thermograms of PANI-g-EPVA nanocomposite films at different aniline contents. (**c**) DTG thermograms of PANI-g-EPVA nanocomposite films at different aniline contents: a–0%, b–20%, c–30%, d–40%, e–45%. (**d**) Storage modulus (G′) and loss modulus (G″) as a function of temperature for PANI-g-EPVA nanocomposite films at different aniline contents: a–20%, b–30%, c–40%.

Conductivity measurements also certify that the electrical conductivity increases from 1.46 × 10^−4^ to 20.83 S/cm with increasing the aniline content from 5% to 45%, as illustrated in Fig. 5b. The electrical conductivity corresponds to 7.3 S/cm when only 30% PANI is incorporated into the composites, while the conductivity of *in situ* PVA/PANI composite is only 2.81 S/cm. The tensile strength of PANI-g-EPVA film increases from 35 MPa to 64 MPa, however, the tensile strength presents a declining trend when the aniline content is greater than 30%. When the aniline content is lower than 30%, the PANI is able to homogeneously distribute in the PANI-g-EPVA nanocomposite on nanometre scale and thereby functionalize as crosslinking points to increase the molecular interactions due to its high polarity, leading to the increase in tensile strength (Figs 2a and 6a). However, further increase in aniline content from 30% to 45% doesn’t strenghten, but even leads to inferior tensile strength. It can be attributed to the formation of PANI aggregation (in Fig. 6a) inside the nanocomposite film, which can destroy the original crosslinking structure and produce inhomogeneous film with defects, resulting in stress concentration and the decrease of tensile strength.

SEM morphology (Figs 2a and 6a) demonstrates that smooth and uniform PANI-g-EPVA nanocomposite film can be obtained when the aniline content is 30%, which is entirely different from the reported research^30^,^50^. In general, PANI aggregations tend to gather on the composite film surface to form inhomogeneous surface^30^,^50^. Chen reported that the original structure of phosphorylated PVA disappeared with the incorporation of PANI, and a large amount of the crystals werefound on the surface of PANI/PVA composites^30^. Whereas the PANI-g-EPVA displays the similar morphology as pure EPVA. This phenomenon further proves that graft polymerisation of PANI into EPVA chains is an effective approach to promote the uniform distribution of PANI in EPVA matrix and the chemical bonds between EPVA and PANI can effectively prohibit the migration of PANI onto the surface of composite films.

To further study the phase behaviour and thermal behaviour of PANI-g-EPVA nanocomposites, the thermogravimetry (TG) and differential thermogravimetry (DTG) thermograms of composite films areinvestigated, as illustrated in Fig. 6b and c. The thermal stability is estimated by the degradation temperature at 10% weight loss (D_0.1_) and 50% weight loss (D_0.5_). The pure EPVA undergoes three main degradation steps. The peak at 50–150 °C is ascribed to the elimination of water molecules, the peak at 250–370 °C is due to the breakdown of side groups, and the peak at 370–480 °C is attributed to the breakdown of mainchain^30^,^51^,^52^. With the incorporation of 30% aniline, only one main degradation peak is observed, demonstrating the good compatibility between EPVA and PANI. Withthe incorporation of 30% PANI, D_0.1_ of PANI-g-EPVA increases from 271 to 313 °C, D_0.5_ increases from 320 to 359 °C, the thermal stability of EPVA can be greatly improved. However, the thermal stability of PANI-g-EPVA is weakened when the aniline content exceeds 30% due to the weakened molecular interactions caused by phase separation, whereas the degradation temperature is still higher than that of pure EPVA.

Figure 6d shows the temperature dependence of the storage modulus and tan δfor PANI-g-EPVA nanocomposite films. The storage modulus is an indicator of the polymer stiffness, and the temperature at maximum tan δis defined as the glass-transition temperature (Tg)^53^. It is found that the storage modulus increases with the incorporation of 30% PANI, indicating an increase in the rigidity of composite film^54^. Since the homogeneous distribution of PANI in PANI-g-EPVA nanocomposites is beneficial for the formation of more uniform network and enhances molecular interactions more effectively, and free PANI inside nanocomposites can also functionalise as a nanoreinforcing agent and crosslinking points in the nanocomposites. It has been demonstrated that enhancing the interaction between nanofillers and polymer matrix is able to increase the mechanical and thermal properties^55^,^56^. However, the storage modulus greatly decreases when the aniline content increases to 45%, owing to the weakened intermolecular interaction. It is also observed that the Tg peak shifts to higher temperature and the intensity of tan δ decreases as increasing the aniline content. It indicates that the movement of polymer chains is restricted with the incorporation of PANI.

### Surface coating of PANI-g-EPVA dispersion on cellulose fibre paper

**Figure 7:**
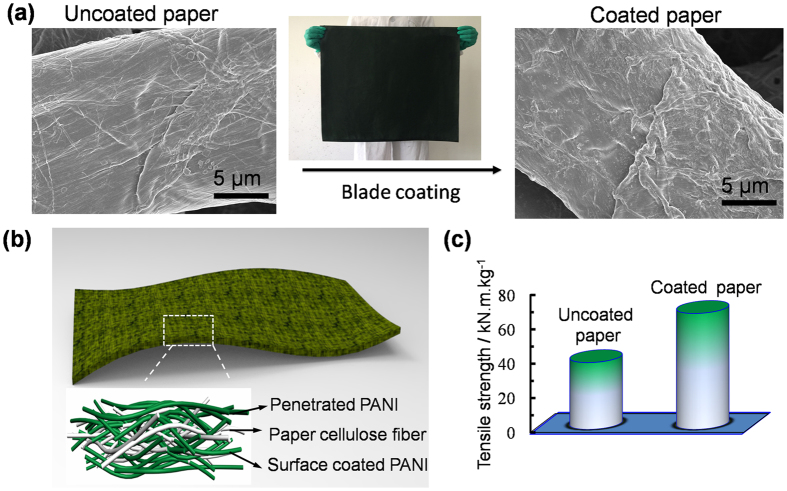
The morphology (**a**), model (**b**) and mechanical property (**c**) of uncoated and coated paper with PANI-g-EPVA.

The as-prepared PANI-g-EPVA dispersion can be directly used as the conductive ink or coating for cellulose fibre paper to prepare conductive paper, which is suitable for scalable production (Fig. 7a). Due to the porous characteristic of cellulose fiber, the PANI-g-EPVA dispersion is able to penetrate into the fiber to form 3D conductive networks in cellulose fiber paper, the corresponding model is shown in Fig. 7b. The electrical conductivity of conductive paper reaches 1.45 S/cm when the PANI-g-EPVA 3 is adopted. It is also worthy to note that the tensile strength of conductive paper increases from 38.9 to 67.5 KN. m. Kg^−1^. In our previous reported study, the mechanical property of conductive paper prepared through *in situ* chemical oxidation polymerisationis generally inferior to the original paper, owing to the acid hydrolysis and the weakened interaction among fibres^57^,^58^. Therefore, surface coating is an effective methodology to simultaneously improve the conductivity and mechanical property of conductive paper.

## Conclusion

Graft polymerization of aniline into epichlorohydrin modified PVA chain is an effective approach to prepare environmental waterborne polyaniline nanocomposites with high stability, conductivity and mechanical properties. The preparation process have been investigated by FTIR, NMR and rheology. The as-prepared PANI-g-EPVA dispersion keeps stable after storage even after one year. In comparison with physical blend and *in situ* polymerisation, more uniform and smooth film can be obtained, and the compatibility between PANI and EPVA is also greatly improved. The electrical conductivity of PANI-g-EPVA film reaches 7.3 S/cm when only 30% PANI is incorporated into the composites. The tensile strength, modulus, and thermal stability also get significant improvement. It can be concluded that building chemical bonds between PANI and other polymer matrix is an effective approach to improve the dispersibility and distribution of PANI in waterborne polymer dispersions, as well as the dispersion stability, and thereby produce high-performance nanocomposites. The as-prepared PANI-g-EPVA dispersion can be directly used as the conductive ink or coatings for cellulose fibre paper to prepare conductive paper with high conductivity and mechanical property, which is also suitable for large scalable production. Besides, the as-prepared waterborne PANI dispersion can be also utilised as conductive ink or coatings for other substrates, such as glass, metal and plastics. Moreover, the present strategy to synthesise waterborne PANI nanocomposites could be easily extended to other polymer matrix and be available for other conducting polymers, such as polypyrrole.

## Methods

### Materials

Poly(vinylalcholol) (PVA:P_n_ = 0588 ± 50, M_w_ = 19800–24200) with 88% alcoholysis degree was obtained from Shanghai Yingjia Industrial Development Co., Ltd. Aniline was purchased from Shaanxi Hongyan Chemical Company, China, and purified by double distillation under reduced pressure before use. Ammonium peroxydisulftate (APS, 99.9%), hydrochloric acid (HCl), epoxy chloropropane (ECIP, 99.9%) and potassium hydroxide (KOH, 99.9%) were all analytical grade and supplied by Tianli Chemical Agents Company, China.

### Preparation of PANI-graft-PVA dispersion

50 g PVA solution (20%, w/w) was introducedintoa 250 ml three-necked flask, and the pH value was adjusted to 11 with KOH solution. Then4 g ECIP was added to initiate nucleophilic substitution reaction between ECIP and PVA, and epoxy groups were incorporated into PVA side chain. The reaction was kept at 80 °C for 30 min. Afterwards, the pH value was subsequently adjusted to 7, and PVA with epoxy side chains (EPVA) was obtained. Subsequently, aniline of different content was added into the EPVA solution. The reaction between -NH_2_ in aniline and epoxy groups in EPVA was conducted at 80 °C for 2 h, and then EPVA-aniline was obtained. Afterwards, the reaction system was placed in an ice-water bath, and the pH value was adjusted to 2 with HCl solution. The chemical oxidation polymerisation of aniline was initiated with APS addition. The reaction was maintained for 24 h, and green PANI-graft-EPVA (PANI-g-EPVA) dispersions were thereby obtained. The preparation procedure and optical photos of PANI-g-EPVA dispersions and films were shown in Fig. 1. In addition, *in situ* PVA/PANI dispersion was prepared by chemical oxidation polymerisation of aniline in unmodified PVA, the optical photos of *in situ* PVA/PANI were also shown in Fig. 1. The low-molecular-weight compounds inside dispersions were removed by exhaustive dialysis in a dialysis bag against 0.2 M HCl.

### Preparation of composite films

The composite films were prepared by casting dispersions into PTFE plate and dried at room temperature for 3 days to obtain conductive composite films.

### Characterisations

The products were extracted with acetone in a soxhlet apparatus for 24 h to remove free PANI and physically absorbed PANI and thereby pure PANI-g-EPVA was obtained. The carbon, nitrogen and hydrogen elemental contents of pure PANI-g-EPVA were determined using a Elemental Analyzer (Elemeraor, Vario EL **III**, Germany). Grafting efficiency (GE) was used to evaluate the graft polymerization of aniline onto EPVA. GE was defined as the ratio of N content by elemental analyzer to the N content calculated by monomer charged. Fourier transform infrared (FTIR) spectra were recorded on an FTIR spectrometer (Bruker Optics Vector-22, Germany) at frequencies of 4000 to 400 cm^−1^ at a 1 cm^−1^ resolution, signal-averaged over 32 scans, and baseline-corrected. The ^1^H-NMR spectra were determined by a nuclear magnetic resonance spectroscopy (NMR, Bruker Avance 400 MHz, Germany). D-Substituted dimethyl sulfoxide was used as a solvent, and tetramethylsilane was used as the internal standard. The morphology of the colloidal particles in dispersion was observed by transmission electron microscopy (TEM, Hitachi S570, Japan) with phosphotungstic acid as a staining agent. The surface morphology of the composite films was observed with an scanning electron microscope (SEM, Hitachi S-4800, Japan). All of the samples were coated with gold in vacuo before SEM observation. The particle size and distribution values of the dispersions were analysed by dynamic light scattering (DLS, Malvern Zetasizer Nano-ZS ZEN 3500, United Kingdom). The rheological properties of dispersion were analysed in an American TA Instrument AR2000ex Rheometer. All tests for dispersion were carried out using DIN concentric cylinders geometry. Samples underwent a constant shearing treatment (1 s^−1^ for 10 min) prior to analysis to remove history effects. The steady measurements were carried out over a range of shear rate from 0.1 to 100 s^−1^ at 25 °C. The time sweep measurements were done at 80 °C with constant stress and frequency of 0.5 Pa and 6 Hz, respectively. UV-Vis absorption spectra were carried out by using a UV-vis spectrophotometer (Shimadzu UV-265FW, Japan) at room temperature, with scan speed of 40 nm/min, integration time of 2 s and bandwidth of 1 nm. The surface resistivity was determined with a four-point van der Pauw method with a Keithley 237 high-voltage source and a Keithley 2010 multimeter. The surface resistivity was measured by a standard four-probe method. The tensile strength of the PVA/PANI films was evaluated on a TS2000-S universal testing machine (Scientific and Technological Limited Co., High Iron, Taiwan). The measurements were repeated at least five times. Thermal analysis of the samples was performed on a thermogravimetric analyser (TGA, Q500, America) under a nitrogen atmosphere in the temperature range of 25–650 °C with a heating rate of 10 °C/min. Dynamic mechanical analysis was carried out on a Q800 dynamic mechanical analyser (TA Instruments Co.) at a frequency of 2 Hz and heating rate of 3 °C/min over a temperature range from −30 °C to 100 °C.

## Additional Information

**How to cite this article**: Wang, H. *et al*. Facile approach to fabricate waterborne polyaniline nanocomposites with environmental benignity and high physical properties. *Sci. Rep.* **7**, 43694; doi: 10.1038/srep43694 (2017).

**Publisher's note:** Springer Nature remains neutral with regard to jurisdictional claims in published maps and institutional affiliations.

## Supplementary Material

Supporting Information
