# Supplementary material for: Facile approach to fabricate waterborne polyaniline nanocomposites with environmental benignity and high physical properties
Source: Sci Rep. 2017 Mar 6;7:43694. doi: 10.1038/srep43694 (PMC5337951; doi:10.1038/srep43694)
Supplement: Supporting Information [file srep43694-s1.doc]

**Facile approach to fabricate waterborne polyaniline nanocomposites with environmental benignity and high physical properties**

Haihua Wang*^1^, Huan Wen^1^, Bin Hu^2^, Guiqiang Fei^1^, Yiding Shen^1^, Liyu Sun^1^, Dong Yang^1^

^1^Key laboratory of Auxiliary Chemistry & Technology for Chemical Industry, Ministry of Education, Shaanxi University of Science and Technology, Xi’an, Shaanxi, 710021, China.

^2^Wuhan National Laboratory for Optoelectronics (WNLO), School of Optical and Electric Information, Huazhong University of Science and Technology (HUST), Wuhan 430074, PR China. Correspondence and requests for materials should be addressed to H.H.W (email: wseaflower@126.com)

**
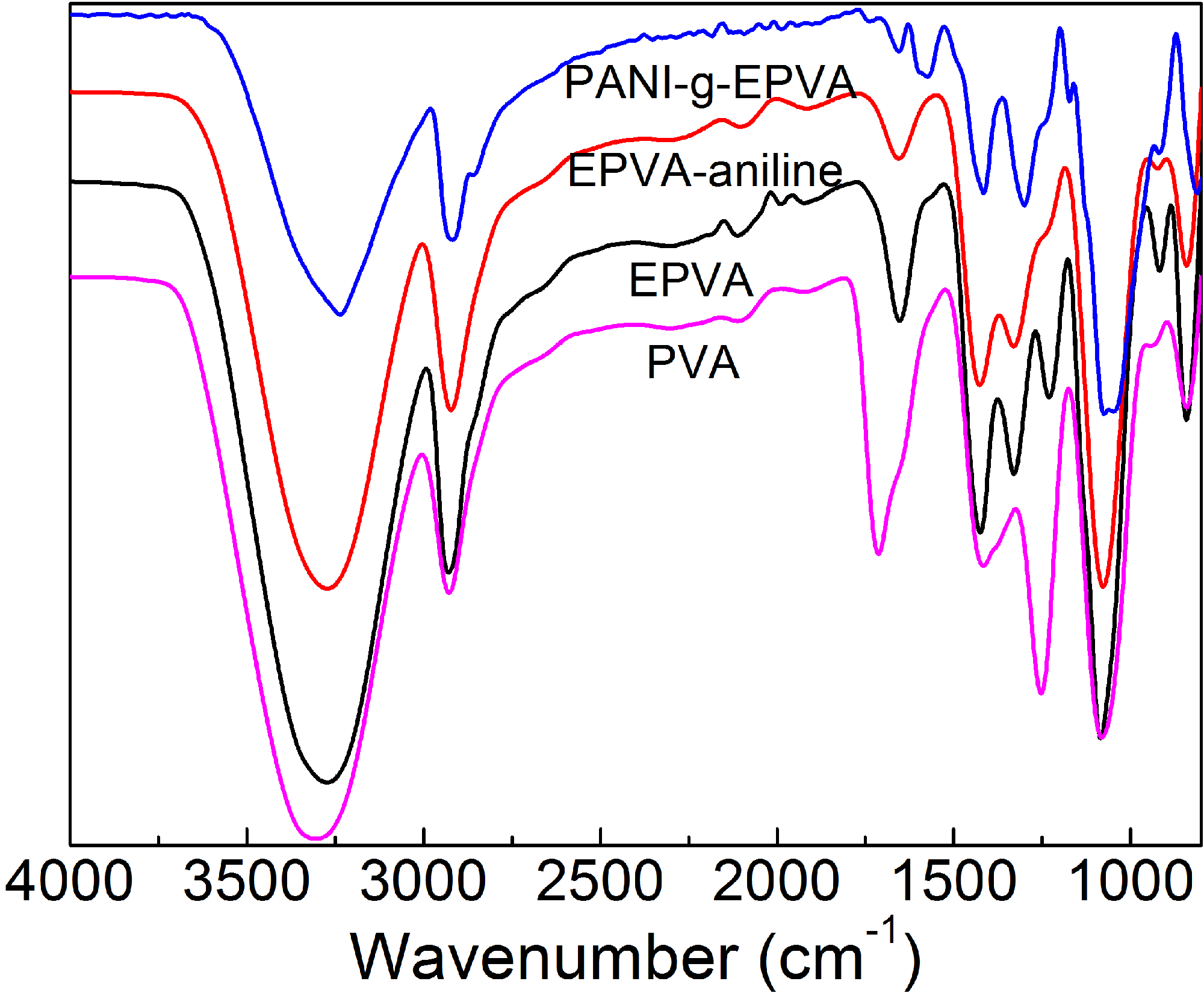
**

**Figure S1.** FTIR spectra of the pure PVA, EPVA, EPVA-aniline and PANI-g-EPVA at 30% aniline content.


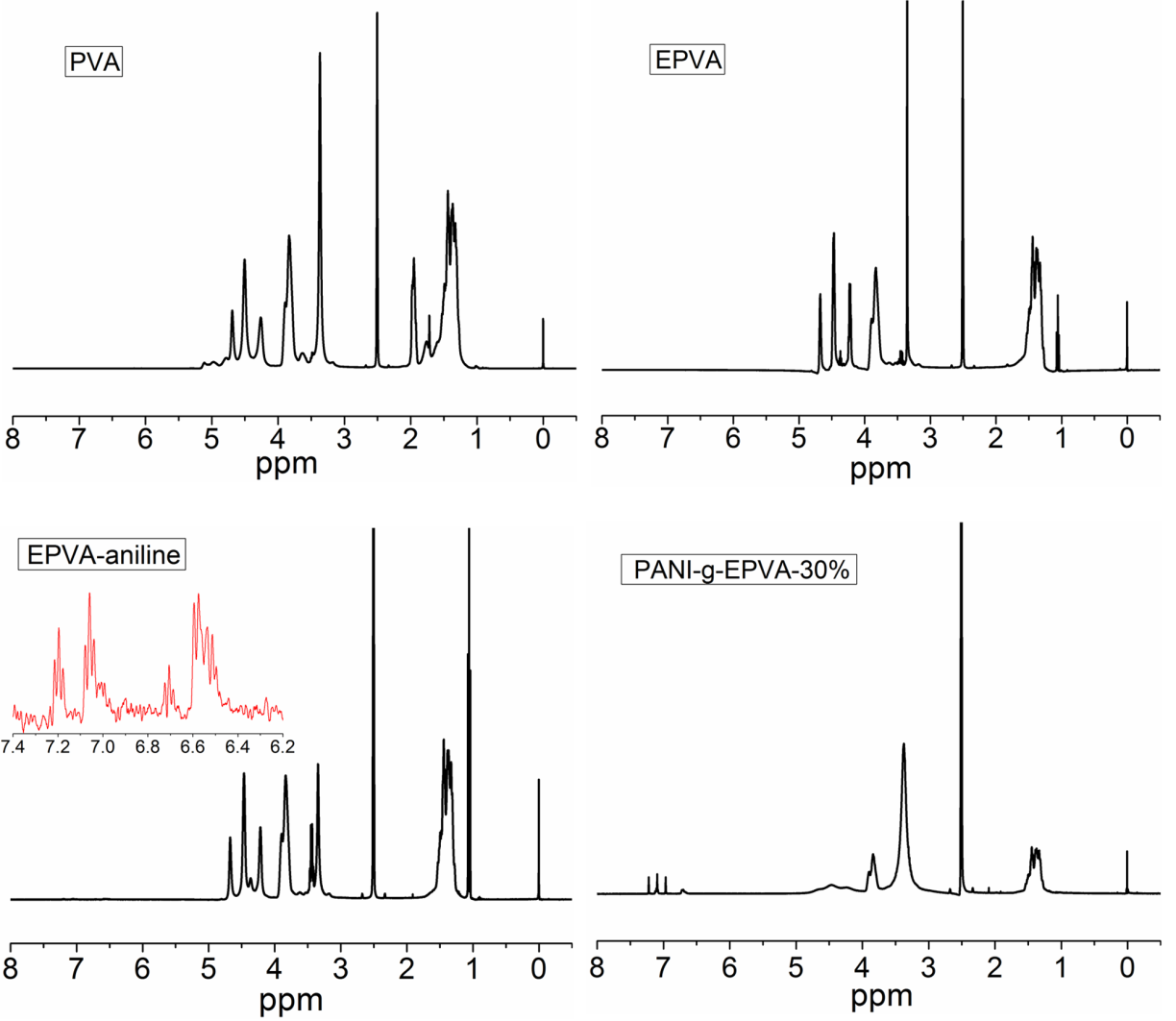


**Figure S2.** NMR spectra of the pure PVA, EPVA, EPVA-aniline and PANI-g-EPVA.

Table S1 Elemental analysis and graft efficiency (GE) of PANI-g-EPVA nanocomposites prepared at different aniline content

| Sample | C | H | O | N | N | GE(%） |
| --- | --- | --- | --- | --- | --- | --- |
|  | Test value | | | | Theoretical value |  |
| PANI-g-EPVA-20% | 50.05 | 9.157 | 38.93 | 1.863 | 3.544 | 52.50 |
| PANI-g-EPVA-30% | 47.89 | 8.513 | 41.01 | 2.587 | 5.316 | 48.66 |
| PANI-g-EPVA-40% | 49.67 | 8.280 | 39.08 | 2.970 | 7.088 | 41.90 |


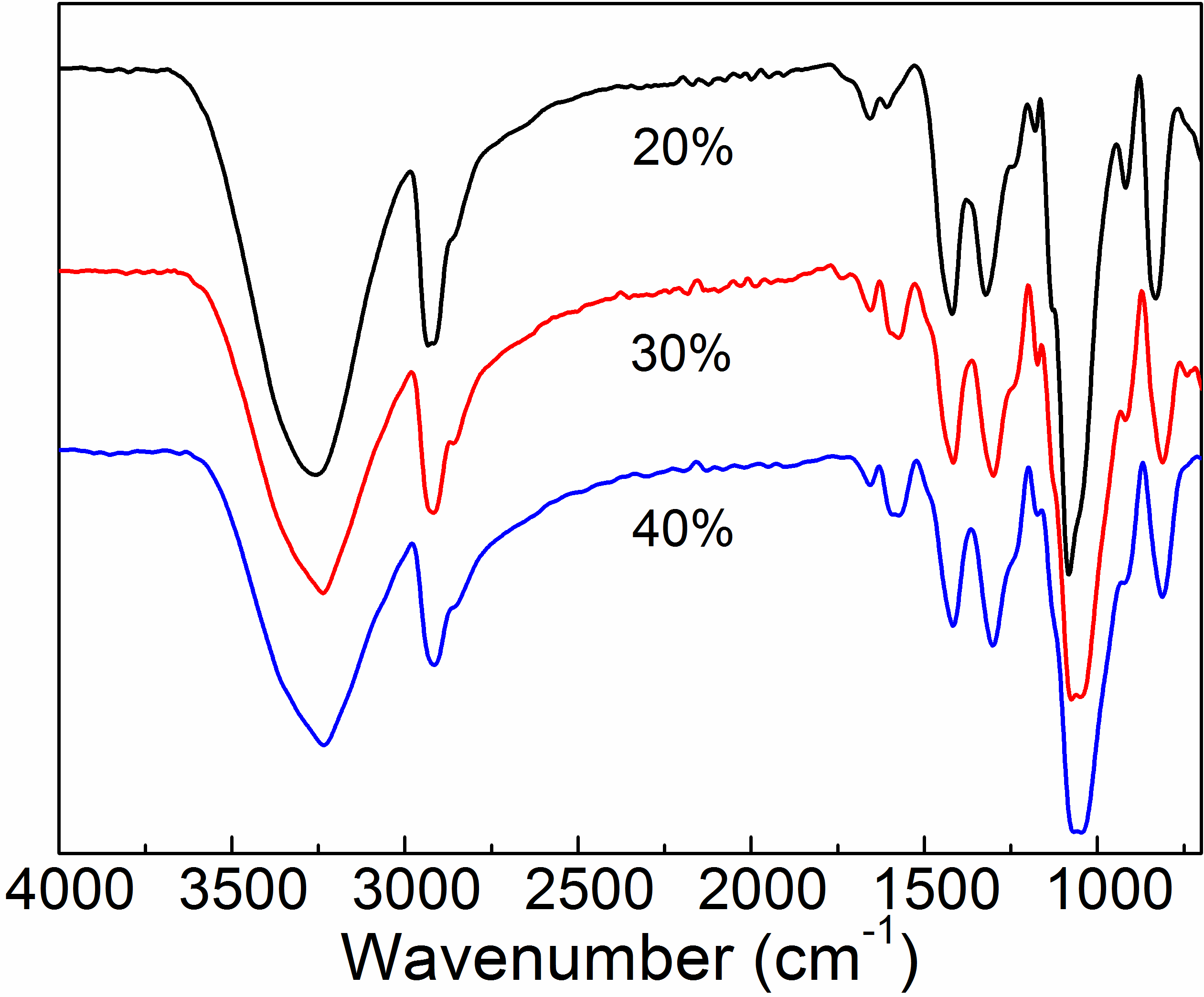


**Figure S3.** FTIR spectra of PANI-g-EPVA prepared at different aniline content.


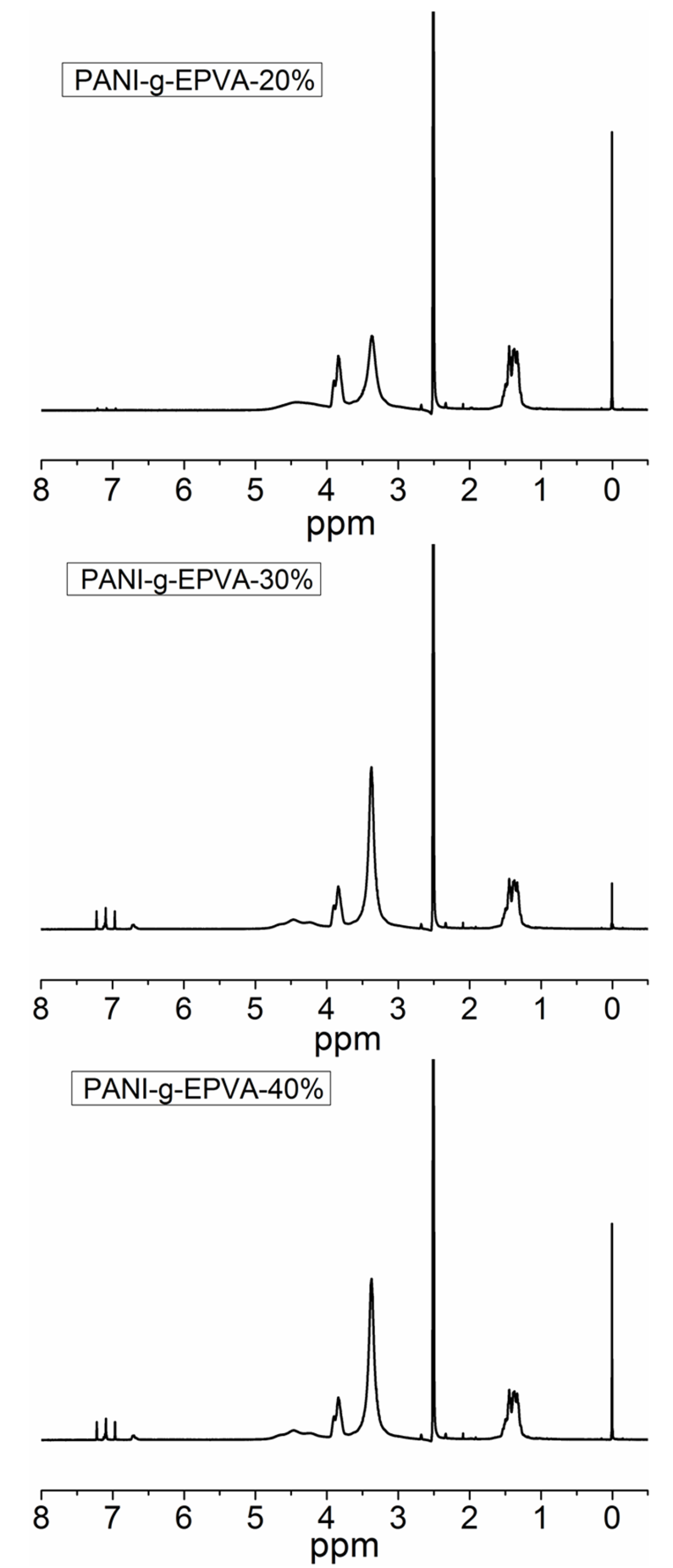


**Figure S4.** NMR spectra of PANI-g-EPVA prepared at different aniline content.

**Figure S5.** Average particle diameter and distribution values of the PANI-g-EPVA dispersions at different aniline content (A) 20% (B) 25% (C) 30% (D) 35% (E) 40% (F) 45%
